# Depressive symptoms, anxiety, and quality of life of Japanese women at initiation of ART treatment

**DOI:** 10.1038/s41598-021-87057-6

**Published:** 2021-04-06

**Authors:** Tsuguhiko Kato, Makiko Sampei, Kazuki Saito, Naho Morisaki, Kevin Y. Urayama

**Affiliations:** 1 Department of Social Medicine, National Center for Child Health and Development, 2-10-1 Okura, Setagaya-ku, Tokyo, 157-8535 Japan; 2 Department of Pediatrics, Perinatal and Maternal Medicine (Ibaraki), Graduate School, Tokyo Medical and Dental University, Tokyo, 113-8519 Japan; 3 Graduate School of Public Health, St. Luke’s International University, 3-6-2 Tsukiji, Chuo-ku, Tokyo, 104-0045 Japan

**Keywords:** Quality of life, Epidemiology, Reproductive signs and symptoms

## Abstract

Assisted reproductive technology (ART) treatment accounted for 6% of total births in 2017 and is increasing which places Japan among the top worldwide in number of treatments performed. Although ART treatment patients often experience heavy physical and psychological burden, few epidemiologic studies have been conducted in Japan. We examined mental health and health-related quality of life (QOL) among women at early stages of treatment. We recruited 513 women who have initiated ART treatment, either in-vitro fertilization or intracytoplasmic sperm injection, from four medical facilities in the Tokyo area and through web-based approaches. At baseline, we collected socio-demographic information and assessed depressive symptoms, anxiety, and QOL. Descriptive analyses were performed overall and stratified by factors such as age. Mild depressive symptoms or worse, assessed with Quick Inventory of Depressive Symptoms, were observed among 54% of participants. Mean score for State-Trait Anxiety Inventory was 52 with a standard deviation of 11 for the state, and 39% were categorized as high anxiety. QOL results, assessed with SF-12, showed the same negative tendency for social functioning and role (emotional), while general health and physical functioning were consistent with the national average. Young participants appeared to suffer mentally more than older participants (*p* < 0.01 for depressive symptoms). Our findings suggest that patients may be at high risk of depressive symptoms, high anxiety, and low QOL even from the early stages of ART treatment.

## Introduction

The low fertility trend has continued over a half century in Japan, and the total births per year have drastically declined, from 2.09 million in 1975 to 0.86 million in 2019^1^. By contrast, the number of births by assisted reproductive technology (ART), such as in-vitro fertilization (IVF) and intracytoplasmic sperm injection (ICSI), have increased steadily during the past 20 years from 12,274 in 2000 to 56,617 in 2017, currently accounting for about 6% of total births per year in Japan^2^. In 2017, about 450,000 treatments were performed, placing Japan at the top worldwide in terms of utilization frequency. Delayed childbearing among Japanese couples is likely to be a main driver for the increase, but the prevalence of infertility, defined as not becoming pregnant after 1 year of unprotected sexual intercourse on a regular basis, remains unknown in Japan due to absence of epidemiological studies.

Medical cost of ART treatment is often very high^3,4^, in which treatment over one cycle costs between 300,000 and 500,000 Yen (approximately 3000–5000 US dollars) or more in Japan according to a 2018 survey^5^. This is an out of pocket expense because medical insurance is not applicable to ART in Japan. Government financial assistance has been available and expanded from 2021, removing household income threshold in response to high demands. Despite the high cost of ART treatment, the reality is that having a child still may not be the outcome: 16% (3555 births/21,939 embryo transfers) success with conventional IVF and 24% (46,396 births/194,415 embryo transfers) success with frozen embryo transfer according to 2017 national data^2^.

In addition to the high cost, ART treatment is known to place heavy burden on physical and mental health, particularly in women^6–8^. In other developed countries, infertility treatment is also on the rise, and numerous epidemiologic studies have examined the negative impact of infertility treatment on mental health and health-related quality of life (QOL). These studies showed that treatment is often associated with high levels of stress and distress and lower QOL among the patients^9–11^. Also, studies have shown that the stress from the treatment may be associated with discontinuation of treatment^12–14^. However, it is still inconclusive whether there is a relationship between stress levels and achieving pregnancy^15–17^.

Despite Japan being number one in the world in terms of number of ART treatments, epidemiologic studies investigating physical and mental health of patients undergoing infertility treatment remain scarce. To our knowledge, there are only three studies published in international journals^18–20^, and no studies have exclusively focused on ART patients. To address this gap, we have initiated a prospective cohort study of women receiving ART treatments; in this report, we introduce the design and methodological features of this ongoing study, and describe the depressive symptoms, anxiety, and QOL, as well as socio-demographic characteristics of women who are in early stages of ART treatment.

## Method

### Study population and recruitment

Women at early stages of ART treatment were enrolled into our cohort study. The eligibility to participate in this study included patients who were either, (1) starting the ART treatment (IVF or ICSI) with no prior experiences, or (2) had started the treatment and retrieved oocytes once or twice at the time of recruitment. We employed two methods of recruitment for this study, one of which was through clinicians at medical facilities. Participants were recruited at four medical facilities in Tokyo and Kanagawa prefectures, which offer infertility treatment. The clinicians distributed a study envelope that consisted of a description of the study, consent form, and baseline survey to eligible patients at the initial counseling session. Those willing to participate filled out the consent form and survey and returned them using an enclosed envelop with the return address and postage. A total of 74 participants were recruited from the medical facilities. The second approach to recruitment comprised the use of a study website which was advertised through social networking services and media-related outlets. Those who visited the website and had interest in participating registered their mailing address, and a study envelope was mailed to them. A total of 439 from 550 registrations participated (80% participation rate). We included those with children as long as they had no experience with ART in prior pregnancies and excluded those who were already pregnant as a result of treatment. The total number of participants was 513, and informed consent to participate in this study was obtained from each participant. Reported content followed the STROBE checklist for cohort studies. This study was performed in accordance with the Japanese Ethical Guidelines for Medical and Health Research Involving Human Subjects, and an approval from ethics committee for this study was obtained at the National Center for Child Health and Development (No. 1993).

### Follow-up

Participants are followed for 1 year during which time a series of six questionnaires are administered sequentially every 2 months in order to evaluate the trajectories of depressive symptoms, anxiety, and QOL as multiple cycles of ART treatment are pursued (i.e., oocyte retrievals and embryo transfers). The number of oocyte retrievals and embryo transfers were ascertained at the baseline survey and at follow-up. Participants are followed up to 1 year or until time of withdrawal or loss to follow-up, and are censored at the point of achieving pregnancy.

### Main outcomes

The main interests of this study are depressive symptoms, anxiety, and QOL among women who begin ART treatment. We used QIDS (the Quick Inventory of Depressive Symptomatology-Self report) to assess depressive symptoms, STAI (the State-Trait Anxiety Inventory) to assess anxiety, and SF-12 to assess QOL. Using the total score for QIDS, we created a five-category variable^21^: none, mild, moderate, severe, and very severe. Using the total scores for state and trait, each was categorized into 5 levels where 1 represented the lowest anxiety level and 5 was the highest; we defined categories 4 and 5 as high anxiety^22^. Based on the SF-12 responses, scores for 8 sub-scales were calculated: physical functioning, role (physical), bodily pain, general health, vitality, social functioning, role (emotional), and mental health. The raw scores were transformed into norm-based scores with the average of 50 and a standard deviation of 10, in which lower scores indicated signs of low QOL^23^.

### Socio-demographic variables

We collected a range of socio-demographic information: age, educational attainment, employment status such as type of employment and company size, residing prefecture, and income of the women/patients and their partners. Type of employment was grouped into three categories: full-time, part-time, and not working. Part-time included those who worked part-time, were contractual, or self-employed. Not working included unemployed, housewife, and student. Education was classified into three levels: high school graduate or below; vocational school or 2-year college; and 4-year college or graduate school. We also obtained information on height and weight to calculate body mass index, durations for marriage and infertility treatment (e.g., intrauterine insemination), reasons for wanting a child, sources of emotional support, a sub-scale for partner relationship^24^, and method of financing for the treatment. We inquired about the age and sex of children if there were any and the method of their conception (i.e., naturally conceived or not).

Despite the increasing popularity of ART treatment, characteristics of patients remain unknown. To further understand socio-demographic characteristics of ART patients in relation to the general population, we utilized 2016 data from the Comprehensive Survey of Living Conditions, comprising a nationally representative sample of Japanese families conducted by the Japanese Ministry of Health, Labour and Welfare, for comparison^25^. From the national data, we extracted married women between the ages of 20 and 45 who do not have children (n = 7468).

### Statistical analysis

We performed descriptive analysis to examine the proportions across categories of QIDS and STAI and average scores for the SF-12 sub-scales among the participants. Additionally, we stratified QIDS and STAI categories and SF-12 sub-scales by number of oocyte retrievals, number of children, and age categories and assessed whether or not the pattern of depressive symptoms, anxiety, and QOL varied by these factors using chi-square tests and simple linear regression. We treated number of oocyte retrievals and age categories as ordinal in the simple linear regression analysis. A two-sided p value of less than 0.05 was considered statistically significant. All the analysis was done with STATA 13 (Stata Corp., College Station, TX, USA).

## Results

In Table 1, we present socio-demographic information of study participants and of women sampled from a nationally-representative survey of the general population (married women with no children). Compared to the general population, we observed a larger proportion of women in their 30 s in this study where the mean age was 35 years (range 24–46 years). The proportion of full-time employment and working at larger companies was higher in this study than the general population (full-time employment: 48% vs. 37%; working at a company with ≥ 1000 employees: 27% vs. 19%), as was the proportion of highly educated women (65% vs. 27%). Participants were mainly from Tokyo and neighboring prefectures. Although income information was not available for the general population, we expect that study participants likely had higher earnings. Half of the participants noted that the reason for infertility was unknown. There were 28 participants (5%) who had 3 or more experiences with oocyte retrieval at baseline. Although they were not eligible to participate in this study, we retained them for comparison purposes.

**Table 1:** Socio-demographic profile of participants (n = 513), compared with national data (n = 7468).

	Participants				CSLC^a^	
	Women		Men		Women^b^	
	n	%	n	%	n	%
**Age**						
< 25.0	5	1	3	1	288	4
25.0–29.9	54	11	44	9	1401	19
30.0–34.9	177	35	157	31	1660	22
35.0–39.9	208	41	168	33	1651	22
≥ 40.0	69	13	141	27	2468	33
**Employment status**						
Full-time	245	48	444	87	2566	37
Part-time	156	31	67	13	2420	35
Not working	107	21	2	< 1	1,916	28
**Company size**^**c**^						
1–29	103	26	94	19	1233	26
30–99	60	15	69	14	836	18
100–299	36	9	47	9	724	15
300–999	57	14	79	16	744	16
≥ 1000	108	27	171	34	899	19
Public	32	8	42	8	341	7
**Education**						
High school graduate or below	64	13	71	14	2507	39
Vocational school or two-year college	112	22	60	12	2205	34
4-year college or graduate school	334	65	378	74	1725	27
**Income**					n.a	
0	43	8				
1–99	60	12	3	1		
100–199	55	11	3	1		
200–299	58	11	18	4		
300–399	87	17	60	12		
400–499	81	16	92	18		
500–599	53	10	104	21		
600–699	30	6	74	15		
700–799	12	2	52	10		
800–899	11	2	43	9		
900–999	4	1	17	3		
≥ 1000	13	3	39	8		
**Number of children**						
0	454	88			7468	100
1	58	11			0	0
2	1	< 1			0	0
**Residing prefecture**						
Tokyo	178	35			316	4
Kanagawa	64	12			385	5
Saitama	33	6			214	3
Chiba	17	3			163	2
Other	222	43			6390	86
**Reasons for infertility**					n.a	
Known	246	48				
Unknown	267	52				
**Oocyte retrieval**					n.a	
0	130	25				
1	261	51				
2	95	19				
≥ 3	28	5				

*n.a*. not avaiable.

^a^Year 2016 Comprehensive Survey of Living Conditions (national data).

^b^Married women between the ages of 20 and 45 with no children.

^c^Among those who are working.

In Table 2, we present levels of depressive symptoms, anxiety, and QOL among the participants. The mean score for the QIDS was 7.0 (SD 4.7), and the state and trait scores for the anxiety scale were 51.7 (SD 11.3) and 52.8 (SD 11.4), respectively. Categorization of the QIDS score showed that 54% of participants were already showing some signs of depressive symptoms. Anxiety also seemed to be high among the participants (39% for categories 4 and 5 for state). We also observed similar patterns with the QOL sub-scale scores. Norm-based scores showed that social functioning, role (emotional), and mental health aspects were low in particular, while physical functioning and general health were near the average.

**Table 2:** Depressive symptoms, anxiety, and quality of life among participants (*n* = 513).

	Mean or N	(SD) or %
**Depression symptoms (QIDS)**^**a**^	7.0	(4.7)
None	234	46%
Mild	163	32%
Moderate	85	17%
Severe	27	5%
Very severe	4	1%
**Anxiety (STAI)**		
State^a^	51.7	(11.3)
Category 1	34	7%
Category 2	87	17%
Category 3	189	37%
Category 4 (high anxiety)	151	29%
Category 5 (high anxiety)	52	10%
Trait^a^	52.8	(11.4)
Category 1	24	5%
Category 2	34	7%
Category 3	128	25%
Category 4 (high anxiety)	231	45%
Category 5 (high anxiety)	96	19%
Quality of life (SF-12)		
Physical functioning^a^	50.7	(9.4)
Role (physical)^a^	43.5	(11.8)
Bodily pain^a^	44.8	(11.7)
General health^a^	53.5	(9.4)
Vitality^a^	47.0	(9.0)
Social functioning^a^	41.7	(14.3)
Role (emotional)^a^	40.4	(12.0)
Mental health^a^	42.6	(10.6)

^a^Mean and standard deviations.

In Table 3, we present depressive symptoms, anxiety, and QOL stratified by number of oocyte retrievals, number of children, and age categories. We did not observe a clear pattern with the exception of physical role functioning appearing lower among those with more oocyte retrievals. We observed those with one or more children seemed to be doing better mentally. Young participants (i.e., < 30.0) seemed to show higher prevalence of depressive symptoms and higher anxiety and lower QOL scores than older participants.

**Table 3:** Depressive symptoms, anxiety, and quality of life among participants, stratified by number of oocyte retrievals, number of children, and age categories (*n* = 513).

	Number of oocyte retrievals									Number of children					Age categories								
	0		1		2		3 ≥		*p* value	0		1 ≥		*p* value	< 30.0		30.0–34.9		35.0–39.9		≥ 40.0		*p* value
	Mean or N	(SD) or %	Mean or N	(SD) or %	Mean or N	(SD) or %	Mean or N	(SD) or %		Mean or N	(SD) or %	Mean or N	(SD) or %		Mean or N	(SD) or %	Mean or N	(SD) or %	Mean or N	(SD) or %	Mean or N	(SD) or %	
**Depression symptoms (QIDS)**^a^	6.3	(4.7)	7.3	(4.7)	6.9	(4.7)	8.4	(4.9)		7.2	(4.8)	5.5	(4.2)		8.3	(4.3)	7.2	(5.1)	6.7	(4.7)	6.2	(4.1)	
None	72	56%	109	42%	44	46%	9	32%	0.31	196	43%	38	64%	0.05	13	22%	83	47%	101	49%	37	54%	< 0.01
Mild	30	23%	88	34%	35	37%	10	36%		151	33%	12	20%		30	51%	49	28%	63	30%	21	30%	
Moderate	21	16%	47	18%	10	11%	7	25%		78	17%	7	12%		10	17%	30	17%	36	17%	9	13%	
Severe	5	4%	15	6%	5	5%	2	7%		25	6%	2	3%		6	10%	14	8%	5	2%	2	3%	
Very severe	1	1%	2	1%	1	1%	0	0%		4	1%	0	0%		0	0%	1	1%	3	1%	0	0%	
**Anxiety (STAI)**																							
State^a^	51.5	(12)	51.8	(11.2)	51.4	(11.3)	53.2	(8.3)		52.1	(11.3)	48.8	(10.8)		54.9	(10)	52.8	(11.7)	50.9	(11.1)	48.7	(10.7)	
Category 1	12	9%	15	6%	7	7%	0	0%	0.73	28	6%	6	10%	0.36	1	2%	10	6%	14	7%	9	13%	0.08
Category 2	17	13%	47	18%	19	20%	4	14%		75	17%	12	20%		8	14%	27	15%	42	20%	10	14%	
Category 3	50	39%	92	35%	34	36%	13	46%		165	36%	24	41%		19	32%	62	35%	75	36%	33	48%	
Category 4 (high anxiety)	37	29%	83	32%	24	25%	7	25%		137	30%	14	24%		23	39%	58	33%	58	28%	12	17%	
Category 5 (high anxiety)	13	10%	24	9%	11	12%	4	14%		49	11%	3	5%		8	14%	20	11%	19	9%	5	7%	
Trait^a^	52.0	(12)	53.4	(11.3)	51.3	(11.5)	54.9	(9.3)		53.1	(11.4)	49.9	(11.3)		57.1	(10.8)	53.7	(11.7)	51.9	(10.8)	49.0	(11.7)	
Category 1	7	5%	13	5%	4	4%	0	0%	0.31	20	4%	4	7%	0.18	2	3%	8	5%	9	4%	5	7%	0.06
Category 2	11	9%	12	5%	8	8%	3	11%		29	6%	5	8%		1	2%	8	5%	17	8%	8	12%	
Category 3	32	25%	66	25%	28	29%	2	7%		107	24%	21	36%		13	22%	40	23%	57	27%	18	26%	
Category 4 (high anxiety)	55	43%	119	46%	38	40%	19	68%		210	46%	21	36%		25	42%	82	46%	91	44%	33	48%	
Category 5 (high anxiety)	24	19%	51	20%	17	18%	4	14%		88	19%	8	14%		18	31%	39	22%	34	16%	5	7%	
Quality of life (SF-12)																							
Physical functioning^a^	51.3	(8.8)	50.7	(9.4)	50.3	(9.8)	49.6	(11.7)	0.29	50.7	(9.6)	50.8	(8.1)	0.96	51.0	(9.1)	50.3	(10)	51.2	(9)	50.4	(9.7)	0.88
Role (physical)^a^	46.1	(10.7)	42.3	(11.9)	43.9	(12.4)	40.7	(11.4)	0.03	43.2	(11.8)	45.6	(11.6)	0.14	41.0	(12.2)	43.4	(12.1)	44.0	(11.4)	44.4	(11.7)	0.10
Bodily pain^a^	46.7	(11)	43.7	(12.2)	45.5	(11.4)	43.8	(10.2)	0.27	44.3	(11.8)	48.1	(10.8)	0.02	42.0	(12.2)	43.8	(11.8)	46.2	(11.4)	45.0	(11.2)	0.03
General health^a^	53.8	(9)	53.3	(9)	54.1	(9.8)	51.5	(12.3)	0.58	53.3	(9.5)	54.8	(7.9)	0.23	53.5	(9.7)	53.0	(10.1)	53.7	(9)	54.0	(8.3)	0.56
Vitality^a^	47.8	(8.9)	46.3	(9.1)	48.0	(8.9)	47.2	(9.1)	0.94	46.8	(8.9)	48.8	(9.6)	0.11	45.5	(9.2)	46.3	(9.2)	47.8	(9)	48.0	(8.4)	0.04
Social functioning^a^	42.9	(13.4)	41.3	(14.7)	42.0	(14.4)	39.0	(15.1)	0.29	41.3	(14.4)	44.4	(13.8)	0.13	37.6	(14.8)	40.3	(14.5)	43.0	(14)	45.0	(13.7)	< 0.01
Role (emotional)^a^	41.8	(11.5)	39.5	(12.4)	41.7	(11.1)	37.2	(13.2)	0.29	39.7	(11.9)	45.9	(11.1)	< 0.01	37.4	(12.3)	39.6	(11.8)	41.4	(11.9)	41.8	(12.3)	0.02
Mental health^a^	43.6	(11.7)	42.1	(10.5)	43.2	(10.3)	41.1	(11.8)	0.42	42.3	(10.6)	44.9	(10.3)	0.09	38.3	(10)	41.7	(10.1)	43.7	(10.7)	45.4	(10.8)	< 0.01

To calculate p values, we used Chi-square testing for QIDS and STAI (state and trait) and simple linear regression for subscales of SF-12 treating categories as ordinal.

^a^Mean and standard deviations.

## Discussion

In this report, we described the profile of a new longitudinal study aimed at addressing gaps in the current epidemiological literature on the mental health and well-being of women undergoing ART treatment in Japan. We provided a first report focused on depressive symptoms, anxiety, and QOL as well as socio-demographic profiles of women who were starting or had just started ART treatments. Results showed that indicators of mental health already appeared unfavorable at the early stages of ART treatment. Descriptive analysis of the socio-demographic characteristics showed that those who begin ART treatment tended to be in their thirties residing in Tokyo or adjacent prefectures, with high education and full-time employment. Although Japan leads the world in the number of ART treatments performed per year, epidemiologic studies on the mental health and QOL of patients remain scarce. To our knowledge, this study presents one of the first opportunities to demonstrate the mental health status of Japanese women struggling with infertility and starting ART treatment to have a child. High prevalence of poor mental health at early stages of treatment is concerning because high stress has been associated with discontinuation of infertility treatment and may have profound influences on daily lives such as sexual functioning^13,26^.

Aligned with previous findings from Japan and other countries, women who go through ART treatments appeared to be highly stressed, indicated by depressive symptoms, high anxiety, and low QOL^9^. One study conducted in Japan showed that 32% of patients undergoing infertility treatment showed depressive symptoms, based on the Center for Epidemiologic Studies-Depression Scale^27^. In another Japanese study comprising 125 patients receiving ART or other infertility treatments, the average scores of STAI were 46.5 (SD 9.5) for trait and 48.6 (SD 8.6) for state^28^. Matsubayashi et al. showed that scores based on the Hospital Anxiety and Depression Scale and Profile of Mood States were higher among infertility patients than pregnant women^18^. Our results were mostly consistent with these previous findings despite the differences in tools to assess mental health. A study by Ogawa et al., also showed elevated levels of anxiety and depression, but the prevalence appeared higher in older patients which was in contrast with our findings of higher prevalence among younger age groups^20^. To our knowledge, no previous studies have examined QOL among infertility treatment patients in Japan.

We showed in our study that women at early stages of the ART treatment were already showing signs of deteriorated mental health and QOL. Reasons for this tendency remain unknown and are to be examined, but one contributing factor may be the expectation of high treatment cost. The total treatment cost could easily reach up to a million Yen or more as they go through multiple cycles of treatment. Another factor may be related to time management needed to coordinate treatment and work issues. Because patients cannot know the schedule of treatment in advance, and frequent clinic visits are necessary, such time constraints could interfere with work. Japanese workers are known for their long work hours^29^, and the corporate culture may cause logistical difficulties for those working full-time. In our study, half of the women were working full-time.

Half of the participants noted that reasons for infertility were unknown. Thus, they do not know which treatment would be best for them to increase the chance of achieving pregnancy. This uncertainty regarding whether the selected treatment is the most effective for their condition may increase stress and anxiety levels. In Japan, the success rate of achieving pregnancy at each fertility clinic is often not available publicly to the patients, while in other countries such as the United States, government agencies publish summary reports^30^. This lack of information may add to the unforeseeable nature of ART treatment, which is likely to be one of the main sources of stress for patients.

Stratified by number of oocyte retrievals, number of children, and age, we observed certain patterns regarding depressive symptoms, anxiety, and QOL. For example, those with at least one child seemed to be better off mentally than those without. This observation may be influenced in part by the decision-making process of choosing to receive the treatment. Only those with suitable circumstances, such as financial capacity, may decide to receive infertility treatment to have another child. For others, the option may not even be available to them due to sub-optimal circumstances. In addition, for those who already have a child, treatment failures may not be as pressuring compared to those who desperately want a first child. High prevalence of depressive symptoms and low QOL among young participants may be explained by the general perception that age is a major contributing factor of infertility. The need for ART treatment may be perplexing and unpleasant to the young participants. Some may have serious health problems which could lead to infertility and also influence mental health negatively. In addition, because of the seniority system and employment structure in Japan, wages for young people tend to be low and may contribute to additional strain on mental health^31,32^.

Our results indicate the need for mental health care support for infertility treatment patients even at the early stages of the treatment. In other countries, intervention studies have been conducted to reduce distress among infertility treatment patients^33^. However, in Japan, such studies have been scarce despite the popularity of infertility treatment. In a study by Asazawa, it was reported that a partnership support program could reduce psychological distress among women^34^. The Japan Society for Reproductive Psychology has translated a publication by the European Society of Human Reproduction and Embryology, “Routine psychological care in infertility and medically assisted reproduction—A guide for fertility staff” into Japanese, and this translated literature is made available on their website^35^.

It is important to acknowledge certain limitations of this study. Because we could not employ random sampling, our results may not accurately reflect the circumstance of the broad-level population receiving infertility treatment in Japan. There are no national data on socio-demographics of the women who go through ART treatments. We may be overestimating the prevalence of depressive symptoms, high anxiety, and low QOL because it is possible that those who wanted to address the difficulties of treatment may have had a higher likelihood of participation. However, the opposite is also conceivable in which those who were not doing well mentally might have had a tendency not to participate. Although we used validated scales such as QIDS, STAI, SF-12, they were not specifically designed to assess special populations such as those who go through ART treatment. In European countries, scales such as Copenhagen Multi-Centre Psychosocial Infertility Fertility Problem Stress Scales (COMPI-FPSS) and SCREENIVF have been developed and used with infertility treatment patients^36,37^, but these were not available in Japanese except for FertiQOL^38,39^. Scales designed for this special population may be necessary. Finally, it is important to note that this descriptive report is based on baseline data taken from a longitudinal survey; thus, it does not reflect the temporal sequence of events. For instance, we do not have access to the participants’ mental health information prior to their initiation of infertility treatment. However, epidemiologic studies on patients who undergo ART treatment are generally lacking in Japan. As such, we believe that descriptive data presented in the current report is important as basis to understanding the problem and informing the direction of future initiatives. Using data from the follow-up surveys, we plan to examine the trajectory of mental health and QOL and potential risk and protective factors that could influence the trajectory^40^.

## Conclusion

Despite the high demand of ART treatments in Japan, studies examining potential negative effects of the infertility treatment on patients’ body and mind remain scarce. In addition to epidemiologic studies to confirm our findings, intervention studies to alleviate the stress of the ART treatment among patients may be needed.
