# Author Correction: Depressive symptoms, anxiety, and quality of life of Japanese women at initiation of ART treatment

**DOI:** 10.1038/s41598-021-02389-7

**Published:** 2021-11-17

**Authors:** Tsuguhiko Kato, Makiko Sampei, Kazuki Saito, Naho Morisaki, Kevin Y. Urayama

**Affiliations:** 1 Department of Social Medicine, National Center for Child Health and Development, 2-10-1 Okura, Setagaya-ku, Tokyo, 157-8535 Japan; 2 Department of Pediatrics, Perinatal and Maternal Medicine (Ibaraki), Graduate School, Tokyo Medical and Dental University, Tokyo, 113-8519 Japan; 3 Graduate School of Public Health, St. Luke’s International University, 3-6-2 Tsukiji, Chuo-ku, Tokyo, 104-0045 Japan

Correction to: *Scientific Reports* https://doi.org/10.1038/s41598-021-87057-6, published online 06 April 2021

The original version of this Article contained an error in Affiliation 2, which was incorrectly given as ‘Department of Pediatrics, Tokyo Medical and Dental University, 1-5-45 Yushima, Bunkyo-ku, Tokyo, 113-8519, Japan’. The correct affiliation is listed below.

Department of Pediatrics, Perinatal and Maternal Medicine (Ibaraki), Graduate School, Tokyo Medical and Dental University, Tokyo 113-8519, Japan.

The original Article has been corrected.

